# DNA Persistent Length in Solutions of Different pH

**DOI:** 10.3390/ijms27010316

**Published:** 2025-12-27

**Authors:** Nina Kasyanenko, Bolorkhuu Khansetsen, Andrey Baryshev, Petr Sokolov

**Affiliations:** Department of Physics, Saint-Petersburg State University, 199034 Saint Petersburg, Russia

**Keywords:** DNA persistent length, protonation and deprotonation, different ionic strengths

## Abstract

In this study, the changes in the DNA native conformation induced by pH changes in the alkaline and acidic regions were examined. It was shown by the methods of low gradient viscometry and flow birefringence that protonation and deprotonation of nitrogen bases inside the double helix cause a change in the persistent length of DNA. The pK values shift with the change in the ionic strength of the solution (NaCl concentration). The additional charges appearing on the DNA bases are not shielded by counterions from the solution. The increase and decrease in the volume of the DNA coil in solution resulting from protonation and deprotonation of base pairs, respectively, are mainly determined by changes in the persistent length of the macromolecule. The stability of the double-helical conformation of DNA ensures the steadiness of the equilibrium rigidity of this macromolecule. The emergence of charges on the bases, resulting from DNA protonation or deprotonation, weakens and even disrupts the hydrogen bonds between complementary bases. However, at the first stage, this occurs without altering the stacking interactions of base pairs, as reflected in the absorption spectra of DNA and in the stability of the DNA persistent length at different pH levels.

## 1. Introduction

The DNA molecule is an important biopolymer due to its crucial biological functions associated with the storage and transmission of hereditary information. The unique structure of the macromolecule, a double helix with a highly charged hydrophilic sugar-phosphate outer frame and hydrophobic flat heterocyclic nitrogenous bases inside, directly supports these functions. It is the alternation of four nitrogenous bases with surprisingly similar physical and chemical properties that determines the entire diversity of living systems. The double-helix structure of DNA exhibits very high rigidity, which can easily change with partial or complete disruption of the system of hydrogen bonds between complementary bases and can just as easily be restored. These changes play significant roles in the processes of transcription and replication as well as enzymatic activity. Additionally, local variations in the physicochemical properties of the environment, such as pH and ionic strength, influence electrostatic interactions among the charged groups of the macromolecule, greatly impacting its conformational capabilities.

Protonation (the addition of a proton (H^+^) to the ionogenic group of a molecule) and deprotonation (the removal of a proton from it) induced by the variation in pH are very important in biological systems. These processes can change the structure and charge properties of biopolymers, which determine the correct performance of their functions in the cell. For example, the existing tautomeric equilibria of nucleic acid bases [1,2] are controlled by pH. The protonation and deprotonation of the bases have a significant effect on their hydrogen bonding ability and, thus, on the structure of DNA as well as its replication, modification, and repair. The physical properties of DNA and how it is recognized by proteins are also altered by pH. Modification of bases significantly affects their protonation and deprotonation [3]. It is known that a biologically significant intercalated-motif (i-motif) depends on pH [4,5,6]. The pH value changing can also be used in the development of various devices with DNA in new technologies [7,8].

Intracellular pH dynamics regulates cell processes involving DNA, such as proliferation, dysplasia, and differentiation. For example, the pH-regulation of transcription factor-DNA binding was found [9]. In several cell types, apoptosis is associated with intracellular acidification and activation of a pH-dependent endonuclease; the pH-dependent DNA breakage was observed [10]. The pH-responsive DNA motifs have attracted substantial attention due to their biological role in vivo [11]. The pH level in cells influences the structure and function of the DNA. This is because the DNA molecule contains both acidic and alkaline components: phosphate groups and nitrogenous bases. The stability of the DNA double helix in biological systems is observed at a pH of 5–9. The phosphate groups of DNA within this region are completely ionized, releasing protons that leave negative charges on the phosphate groups. These negative charges protect DNA from hydrolysis by repelling nucleophiles that would otherwise hydrolyze it. If the pH value becomes too acidic or alkaline, DNA molecules may denature (melt).

Despite the importance of studying the influence of pH on the course of critical biological processes in vitro, the analysis of conformational changes of DNA in vitro due to variations in environmental conditions (pH, ionic strength, and solvent ionic composition) also requires close attention [5,6,12,13,14,15]. DNA is a unique and essential polymer, possessing a unique structure and enormous charge density. Such research will enrich our knowledge of the physics of biological polymers. Indeed, DNA is an interesting molecule due to both acidic and alkaline components (the acidic phosphate group and alkaline and acidic atomic groups on nitrogenous bases). It should be emphasized that changes in DNA conformation at different pH are induced by the emergence of charges, mainly within the helix.

DNA protonation has been studied in some detail in early research. In solutions of free nitrogen bases, N3 of cytosine (N3C) is the first group to be protonated, followed by N1 of adenine (N1A), and, finally, N7 of guanine (N7G). However, in double-stranded DNA, N1A and N3C are involved in the formation of hydrogen bonds inside the helix, and the weakest proton-acceptor group, N7G, located in the major groove of DNA, is protonated first [1]. Therefore, nitrogenous bases in native DNA are protonated at significantly lower pH values. It has been suggested that a proton from N7 G is transferred to N3C due to the formation of a Hoogsteen pair [16]. Since the protonation of DNA bases redistributes electron density within their heterocycles, one can observe specific changes in the spectral properties of DNA (UV and CD spectra) [17,18]. The deprotonation of DNA at alkaline pH is much less studied. To some extent, this can be explained by the absence of DNA spectral changes at alkaline pH. Two DNA bases have the ability to ionize with pH increases, thymine (O4T) and guanine (O6G), as a result of keto-enol tautomerism [1]. A significant change in pH induces alkaline or acid DNA denaturation, resulting in hyperchromism in the DNA absorption spectrum.

DNA is a very convenient biopolymer for investigators. Unlike proteins or RNA, native high-molecular DNA (more than 3000 base pairs) forms a classic statistical coil in solution with more than 10 statistical segments, and fairly short molecules (less than 200 base pairs) can be considered as rod-shaped structures. The conformation of macromolecules of intermediate length is well described using the persistent (wormlike) chain model. The study of the structure and properties of DNA in aqueous solutions is widely used. Indeed, water is the natural environment for DNA in vivo, and its molecules are practically the components of the secondary structure of the macromolecule. The water molecules from the first hydration shell of DNA are highly ordered, especially in the minor groove [19].

DNA is a highly charged polyelectrolyte: there are two electron charges per 0.34 nm of its length. Traditionally, a distinction is made between short-range electrostatic interactions of nearest charged groups along the chain, which can affect the bending rigidity of the macromolecule, and long-range electrostatic interactions of charged groups spaced along the chain, which determine the polyelectrolyte swelling of polyions. Phosphate groups are charged at pH > 1.5 due to the very low pK value [20]. The existence of ionic groups on nitrogenous bases within the helix can, in certain pH ranges, transform a polyanionic macromolecule into a polyampholyte or a polyanion with an even higher charge density. The pK value in this case depends significantly on the ionic strength of the solution [21].

The value of the persistent length of DNA and its dependence on the ionic strength of the solution had long been the subject of debate. The methods of dynamic, electrical, and magnetic birefringence [22,23,24] allowed us to reach consensus on the high rigidity of DNA in its native state: the persistent length of DNA is about 50 nm under physiological conditions. Single-stranded DNA has a much lower bending rigidity (1–2 nm at different salt concentrations) [25]. The conformation of single-stranded DNA in aqueous solutions is difficult to study due to the spontaneous pairing of hydrophobic bases. Essentially, only the charged phosphates prevent the precipitation of a single-stranded DNA from the water solution. Indeed, in this case, the polymer–solvent interaction is radically different from the case of hydrophilic double-helix DNA. Experiments show that the screening of charged phosphate groups induces the precipitation of denatured DNA at a NaCl concentration of more than 2 M [26]. The dependence of the DNA persistent length on the ionic strength of the solution has been studied previously, both experimentally and theoretically [23,27,28]. Odijk–Sckolnick–Fixman’s (OSF) theory, valid for rigid, highly charged polymers, quite effectively reflects the experimentally observed dependence, according to which the persistent length of DNA varies proportionally to the square of the Debye radius r_D_. The change in the Debye radius reflects the change in the ionic strength of the solution. The change in pH value can induce the appearance of additional charges (positive or negative) on the DNA bases within the helix. How does this change affect the persistent length of the macromolecule? How does the ionic strength of the solution influence this process?

Some non-canonical DNA structures like G-quadruplexes, *i*-motif, and triplexes have been identified in vivo. Their formation and stability are determined by the properties of the environment (pH, ionic strength, etc.). Generally, short oligonucleotides are used in related studies. However, in vivo DNA is a macromolecule. The polyelectrolyte nature of DNA determines its properties and functions. The monitoring of the bending rigidity (persistence length) of DNA with variations in environmental conditions is necessary for the understanding of molecular mechanisms of DNA conformational changes in vivo.

Despite many studies of the persistent length of DNA in solutions with different ionic conditions, the effect of DNA protonation and deprotonation on this conformational parameter has remained unclear. In this study, spectral and hydrodynamic methods allowed us to determine the dependence of the persistent length of DNA and its secondary structure on the appearance of additional negative or positive charges on DNA bases.

## 2. Results and Discussion

### 2.1. DNA Rigidity in Solutions with a Neutral pH Value

High-molecular-weight DNA, as a polyelectrolyte, forms a swollen statistical coil in aqueous solutions with its own counterion, Na^+^. In our experiments, the counterion concentration did not exceed 10−4 M. Typically, a supporting electrolyte (NaCl) is added to DNA solutions to control the ionic strength *I*, which is usually expressed in moles of NaCl.

**Figure 1:**
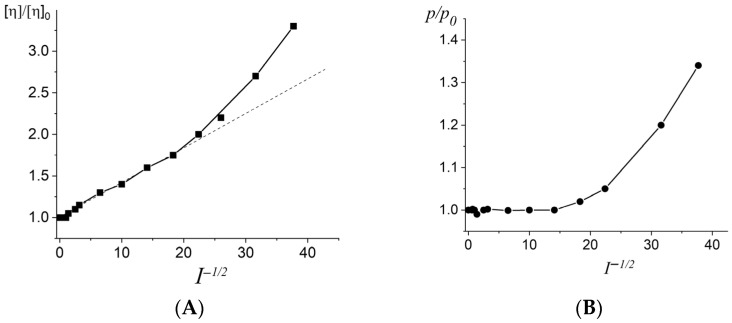
Dependence of the relative change in the intrinsic viscosity (**A**) and in the persistent length (**B**) of DNA *p* on *I*^−1/2^ for DNA with *M* = 9 × 10^6^. Parameters [η]_0_ and p_0_ were defined in 0.15 M NaCl.

The dependence of the intrinsic viscosity of DNA [η] on the volume of the molecular coil and the value of *p* (DNA persistent length) on *I*^−1/2^ for DNA used demonstrates the conformational changes of the macromolecule with the variation in the ionic strength of the *I* solution (*I*^−1/2^ is proportional to the Debye radius). One can see (Figure 1) that an increase in *p* value at *I* < 0.004 leads to curvature of the linear dependence of [η] on *I*^−1/2^. In our studies, we limited the area of ionic strengths to the value of *I* > 0.004 M NaCl. The value of the persistent length in this region can be calculated from the birefringence data. The optical anisotropy of the DNA statistical segment (γ_1_-γ_2_) calculated according to Formula (3) from the reduced birefringence value $\frac{{\left(\Delta n/g\right)}_{g\to o}}{(\eta_{r}-1)\eta_{0}}$ = −(24 ± 2) × 10^−8^ cm·s^2^·g^−1^ gives *p* = (47 ± 3) nm from Formula (4). The average value of the optical anisotropy of DNA base pair Δβ is known [22,23,24] and can be used to find *p* = (450) nm. This value agrees well with existing data (see, for example, the article [15] with the *p* values from various studies cited therein).

### 2.2. DNA Deprotonation in Alkaline Solutions

**Figure 2:**
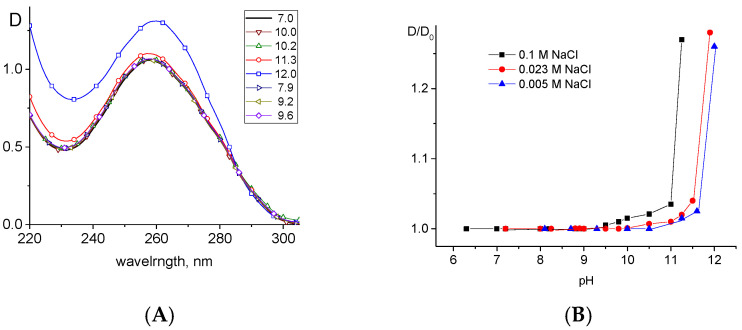
(**A**) DNA absorption spectra (D is the UV absorption of DNA at C(DNA) = 0.005%) at different pH values in the alkaline area (pH values are shown near the lines); (**B**) dependence of the relative change in the DNA absorption D at 260 nm on pH in solutions with different NaCl concentrations (shown near lines). D_0_ is DNA absorption at neutral pH = 7.

Figure 2A shows the absorption spectra of DNA at different pH values in 0.005 M NaCl. As can be seen from the figure, no significant changes in DNA absorption are observed up to pH = 10.2. Hyperchromism appears only at pH > 10.3, which indicates the destruction of base stacking. A sharp increase in DNA absorption without a band shift at pH > 11 indicates an alkaline denaturation of DNA as a result of the deprotonation of bases. The observed result can be explained by the fact that the ionogenic groups of thymine and guanine (O4T and O6G), which lose a proton upon deprotonation, are located outside the heterocycles responsible for the absorption of DNA in the spectral region under consideration. Therefore, one can see only a violation of the stacked structure of the bases, which is responsible for a decrease in their absorption in the double helix compared to their free state. The dependence of the amplitude of the DNA absorption band on pH (Figure 2B) for different NaCl concentrations in DNA solution (0.1 M, 0.023 M, and 0.005 M) indicates that the ionic strength has an effect on DNA deprotonation. Since DNA deprotonation occurs inside the helix, it is unlikely that the change in the shielding of negative DNA phosphates with a varying salt concentration has a direct influence on this process. It should be assumed that with a decrease in the shielding of DNA charges at low ionic strength, the real local concentration of protons near DNA will become higher than the average pH value in the volume of a solution, leading to the result that DNA deprotonation is observed at higher average pH values in decreasing salt concentrations.

**Figure 3:**
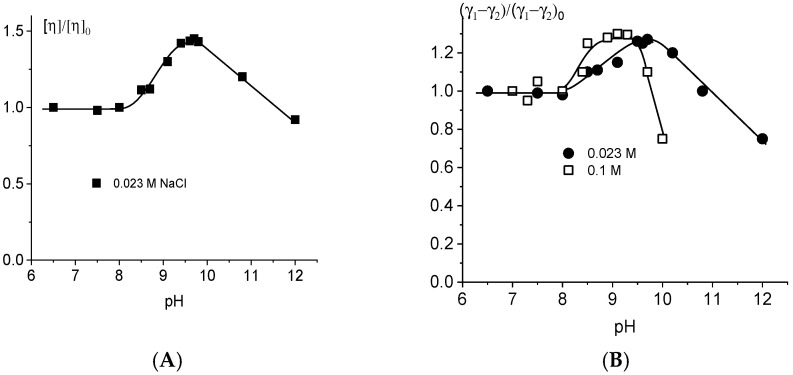
Relative change in DNA intrinsic viscosity [η]/[η]_0_ (**A**) and in the optical anisotropy of DNA statistical segment (γ_1_-γ_2_) (**B**) with alkaline pH value in 0.023 M NaCl and 0.1 M NaCl. Parameters [η]_0_ and (γ_1_-γ_2_)_0_ refer to their values at neutral pH = 7.

Deprotonation induces an increase in the charge density of DNA. Since the charges on the bases inside the helix are not shielded by counterions presented in a solution, one can expect an increase in the persistent length of DNA due to the repulsion of closely located negative charges. Figure 3 demonstrates the influence of the pH value on the intrinsic viscosity of DNA [η] (A) and on the optical anisotropy of the DNA statistical segment (γ_1_-γ_2_) (B). The variation in intrinsic viscosity reflects changes in the volume of the molecular coil of high-molecular-weight DNA in solution. The optical anisotropy of the DNA statistical segment is proportional to the DNA persistent length *p* and to the average difference in the polarizability of the DNA base pair Δβ in the directions along and normal to the helical axis.

One can see similar types of dependencies. Flory’s Formula (2) shows that the change in DNA intrinsic viscosity [η] can reflect the variation in α value (or in the polyelectrolyte swelling of DNA) or in the DNA persistent length *p*. The hydrodynamic length *L* and DNA molecular mass *M* do not change in the experiment. At the same time, an increase in the optical anisotropy of the DNA statistical segment (γ_1_-γ_2_) can be caused by an increase in either the persistent length *p* or in the value of Δβ. However, for DNA in the B form (in water solution), Δβ has a maximum value. It remains to be confirmed whether a change in (γ_1_-γ_2_) unambiguously indicates an increase in the persistent length of DNA upon deprotonation due to an increase in the negative charge density of the macromolecule. A further drop in the value of (γ_1_-γ_2_) with an increase in pH is associated with destabilization of the DNA secondary structure due to the repulsion of negatively charged bases of complementary chains, which ultimately leads to alkaline denaturation of DNA. This is also evidenced by hyperchromism in the DNA absorption spectrum. Therefore, the behavior of DNA optical anisotropy in alkaline pH reflects the growth of the persistent length of a macromolecule. This must necessarily be accompanied by growth in the volume of a molecular coil. Indeed, Figure 3A shows the increase in [η] value within the same pH range.

The contribution of the change in DNA rigidity to the observed increase in [η] value can be calculated. The results showed that the change in the intrinsic viscosity of DNA due to the deprotonation is completely described by the change in its persistent length without taking into account the variation in polyelectrolyte swelling. This can be explained by the fact that deprotonation leads to a change in DNA charge within the double helix. The counterions screen the phosphate groups of the macromolecule. But they cannot penetrate inside the helix to screen the charge on the bases. Indeed, as the experiment showed, the increase in DNA rigidity due to deprotonation of bases does not depend on the ionic strength of the solution (see Figure 3B). The almost imperceptible change in polyelectrolyte swelling upon DNA deprotonation, as opposed to a noticeable change in rigidity, can be explained by the attraction of protons to the macromolecule. This local pH change may also affect charge screening, which determines the excluded volume of the segment responsible for the linear swelling coefficient.

**Figure 4:**
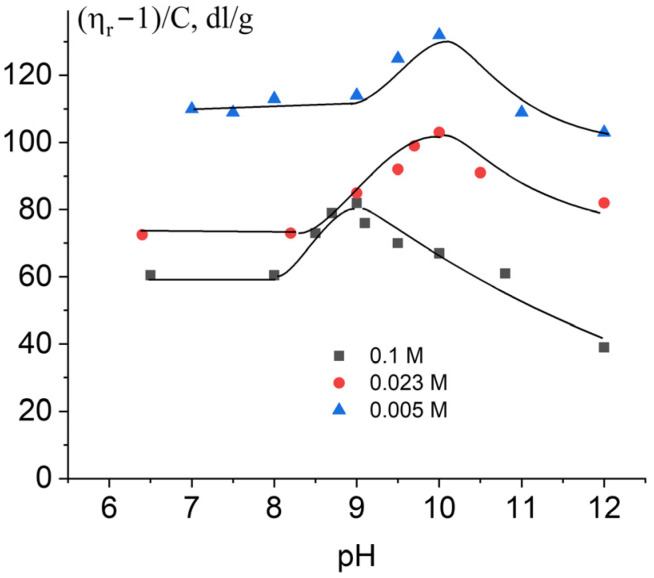
Dependence of the reduced viscosity of DNA solutions on pH in 0.1 M, 0.023 M, and 0.005 M NaCl.

The shift in deprotonation with a change in NaCl concentration is also clearly visible when studying the dependence of the reduced viscosity of DNA solutions on pH (Figure 4). Relative viscosity was measured for one DNA concentration (*c* = 0.0067%) at different ionic strengths of solution (0.1 M, 0.023 M, and 0.005 M NaCl). It is known that only the intrinsic viscosity of polymers can be used for the calculation of conformation parameters of macromolecules. However, an analysis of the dependence of the reduced viscosity of polymer solutions on pH can quite adequately reflect the general course of the dependence of the volume of a molecular coil on this parameter. One can see a similar type of dependence with the shift to the higher pH region with a decrease in NaCl concentration (Figure 4). Let us try to estimate the pK value for DNA in the alkaline pH region as a midpoint of its transition to the deprotonated state. The onset of deprotonation is not easy to determine. The first slight increase in the DNA parameters under study can be taken as the starting point. Usually, the pK value is determined as the midpoint of the DNA transition to the deprotonated state. The middle of the area in the dependences on Figure 3 and Figure 4 from the beginning of the increase in parameters under study to the maximum point for each curve shows the pK value for DNA deprotonation. This value decreases with the increase in salt concentration: pK = 8.5 ± 0.1 in 0.1 M NaCl, pK = 9.0 ± 0.1 in 0.023 M NaCl, and pK = 9.5 ± 0.15 in 0.005 M NaCl. Note that the drop in viscosity at higher pH value can be regarded as a result of destabilization of DNA structure and of the decrease in DNA rigidity preceding the alkaline denaturation of the macromolecule.

It should be assumed that at low ionic strength, with a decrease in the screening of phosphate groups, the local concentration of protons near DNA will become higher than the average value in the volume of a solution, leading to the result that DNA deprotonation starts at higher pH values with a decreasing salt concentration.

### 2.3. DNA Protonation in Acid Solutions

**Figure 5:**
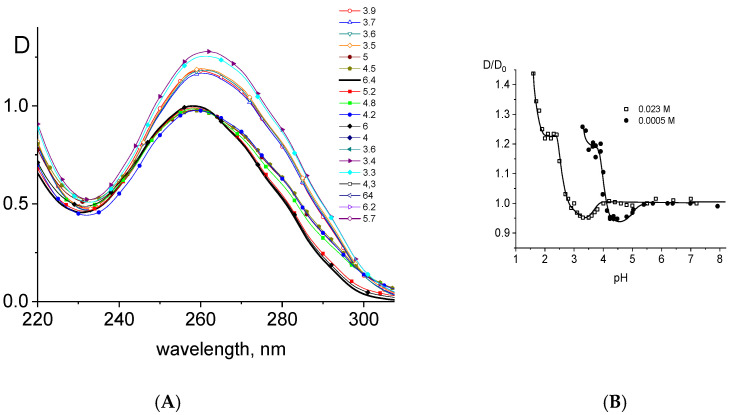
(**A**) DNA absorption spectra at different pH values in the acid area (D is the absorption of DNA in 0.005 M NaCl), pH values are shown near lines, C(DNA) = 0.005%; (**B**) dependence of relative change in DNA absorption at 260 nm on pH in solutions with different NaCl concentrations (shown near lines). D/D_0_ value is explained in the caption to Figure 2.

When considering DNA protonation, it is convenient to use spectral methods, since the bases, as the main chromophores of nucleic acids, directly participate in protonation. Figure 5A shows changes in the DNA absorption spectra with protonation.

When considering DNA protonation, it is convenient to use spectral methods, since the main chromophores of nucleic acids (heterocycles of bases) directly participate in protonation. Processing of these data revealed a well-known result. A slight hypochromic effect with the gradual appearance of a long-wavelength shoulder is observed in the DNA spectrum. At lower pH, a pronounced hyperchromic effect is visible due to DNA acid denaturation. A bathochromic shift in the absorption band is observed after DNA protonation. Figure 5B shows the change in amplitude of the DNA absorption band with decreasing pH in 0.023 and 0.005 M NaCl. One can observe that DNA protonation depends on the salt concentration in the solution, as observed in the alkaline pH region: an increase in NaCl concentration leads to a shift in dependence to lower pH.

The constancy of the DNA absorption in a fairly wide range of acid pH indicates the absence of protonation of the bases and the stability of DNA secondary structure. It is noteworthy that acidic denaturation of DNA is preceded by a noticeable change in its spectral properties. At the early stage of protonation, the double-stranded structure of DNA is not disrupted. The decrease in absorbance at 260 nm and the appearance of the shoulder in the absorption band are usually explained by the protonation of N7 guanine in the DNA major groove [16,20]. The protonation of this group weakens hydrogen bonds between complementary bases. This is followed by the protonation of stronger proton-acceptor groups (N3C and N1A), which are opened as a result of the disruption of hydrogen bonds between complementary bases. Finally, the protonation of DNA bases leads to the disruption of the double-stranded structure and DNA denaturation with hyperchromism in its spectrum. A sharp increase in optical density at a pH below 1.8 can be associated with the attachment of a proton to the oxygen of the phosphate group. Since the charged phosphate group ensures the hydrophilicity of the macromolecule, this transforms DNA from hydrophilic to hydrophobic polymer and leads to partial precipitation of DNA with the appearance of turbidity in the solution and a great increase in its optical density.

**Figure 6:**
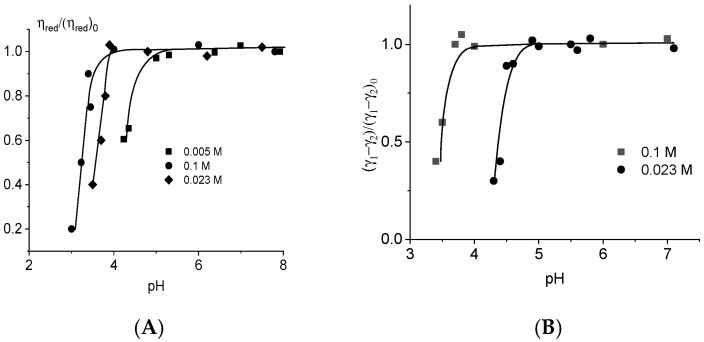
Relative change in the reduced viscosity of DNA solutions (**A**) and in the optical anisotropy of the DNA statistical segment (**B**) with pH at different ionic strengths. Index “0” corresponds to the value of the parameter at neutral pH = 7.

Figure 6 shows the dependence of the reduced viscosity of DNA solutions with different NaCl concentrations on the pH value in the acidic region.

DNA packaging occurs at an acidic pH due to the attraction of positive charges on the bases to negatively charged phosphate groups. It is difficult to separate the changes in the polyelectrolyte swelling and in the persistent length of DNA (long- and short-range electrostatic interactions). At the same time, the optical anisotropy of DNA statistical segments indicates the decrease in DNA rigidity during protonation (Figure 6B).

Thus, in our study, we examined the effect of pH on DNA conformation in solution. The combined use of various methods allowed us to isolate the role of DNA’s persistent length in the observed changes in molecular coil volume while maintaining the macromolecule’s secondary structure. In the future, the study of pH’s influence on conformational transitions will be continued, and a number of issues related to the pH-induced transition from double-stranded to molten DNA will be examined in more detail.

## 3. Materials and Methods

### 3.1. Materials

Commercial protein-free DNA (deoxyribonucleic acid sodium salt from calf thymus D1501, Sigma Aldrich, St. Louis, MO, USA) with the molecular mass of M = 9 × 10^6^, determined from DNA intrinsic viscosity in 0.15 M NaCl, was used. DNA fibers were dissolved in distilled water. Stock solutions containing 0.005 M NaCl were stored at 4 °C. After 5 days, the DNA concentration, molar extinction coefficient, and hyperchromic effect in the DNA absorption spectra during thermal denaturation were determined to verify the nativity of DNA in solutions. The pH value in the DNA solution was changed with 0.01 M HCl or NaOH.

The following conditions were chosen to study DNA protonation and deprotonation. The ionic strength was achieved by introducing a NaCl solution. The electrostatic interactions in DNA solutions are suppressed at NaCl concentrations of more than 1 M NaCl. There is no polyelectrolyte swelling of DNA under these conditions. DNA is a rigid molecule, and its volume effects are not noticeable in solutions corresponding to physiological conditions (0.15 M NaCl). At a salt concentration of 0.023 M, the polyelectrolyte swelling is quite pronounced, and an increase in the volume of the DNA molecular coil is very clearly visible in solutions of low ionic strength (0.005 M NaCl). In our experiments, solutions with very low NaCl concentrations, where the persistent length of DNA increased noticeably, were excluded. Indeed, in this case, the double helix of DNA is not highly stable, making it difficult to isolate the effect of protonation and deprotonation on the conformational parameters of the macromolecule. Usually, the hyperchromism in the DNA absorption spectrum identifies a disruption of the double-helical structure. The eperiments were carried out by hydrodynamic methods under conditions where hyperchromism in DNA absorption spectra did not exceed 4%.

### 3.2. Viscosimetry

The relative viscosity of DNA solutions *η_r_* = *η*/*η_0_* (where *η* and *η_0_* are the viscosities of the solution and the solvent, respectively) was measured at different velocity gradients g in the range of (0.5–2) s^−1^ with a low-gradient rotation viscometer, the principle of which was given earlier [29]. The usage of the *η_r_* value at *g* → 0 gives the reduced viscosity of the DNA solution *η*_red_ = (*η*_red_ − 1)/*c* at different DNA concentrations *c.* The extrapolation of the dependence of *η*_red_ on *c* to a zero DNA concentration provides the DNA intrinsic viscosity value [*η*]:

(1) $$[\eta ]=\underset{c\to o}{lim}\left(\frac{\eta_{r}-1}{c}\right)$$

For DNA with M > 3 × 10^6^, the model of a swelling statistical coil can be used. Both Kuhn’s model of a polymer chain, consisting of freely jointed statistical segments with length *A*, and the model of a wormlike chain with the persistent length *p* can be used in this case. *A = 2p*. For the [*η*] value, Flory’s formula is applicable:

(2) $$[\eta ]=\left(\frac{\Phi {<h^{2}>}^{3/2}}{M}\right)=\Phi \frac{(L2p{)}^{3/2}}{M}\alpha^{3},$$

where Φ is the Flory parameter, *M* is the molecular mass, *L* is the hydrodynamic length of the DNA molecule, and α is the coefficient of linear swelling defining the volume effect, including the polyelectrolyte swelling. The parameter <*h*^2^>^1/2^ (the mean square distance between the ends of the polymer chain) defines the linear size of the molecular coil, <*h*^2^>^1/2^ = *α*(*LA*)^1/2^.

The dependence of the reduced viscosity of a DNA solution on the pH value at a constant DNA concentration was examined. Our study revealed the influence of the pH value on the macromolecule volume, which is determined by the bending chain rigidity (DNA persistent length *p*) and polyelectrolyte swelling α at constant *M* and *L*. The decrease in the reduced viscosity indicates the decrease in the volume of the DNA molecular coil.

### 3.3. Flow Birefringence

The birefringence value Δ*n* for the DNA solution was measured at different velocity gradients *g*. The extrapolation of (Δ*n*/*g*) to *g* = 0 was used to calculate the value of $\frac{{\left(\Delta n/g\right)}_{g\to o}}{(\eta_{r}-1)\eta_{0}}$ which, for DNA, provides the possibility to find the optical anisotropy of the Kuhn statistical segment (γ_1_-γ_2_) (the difference between the polarizabilities of the segment along and normal to the helix axis of the DNA):

(3) $$\frac{{\left(\Delta n/g\right)}_{g\to o}}{(\eta_{r}-1)\eta_{0}}=\;\frac{4\pi }{\;\;45kTn_{s}}\frac{(n_{s}^{2}-1{)}^{2}}{n_{s}}(\gamma_{1}-\gamma_{2}),$$

where *n_s_* is the refractive index of the solvent. The optical anisotropy of the statistical segment gives the product of the number of base pairs in the DNA statistical segment (*S*) and the optical anisotropy of a base pair Δβ along and normal to the axis of the DNA helix:

(γ_1_-γ_2_) = *S*Δβ = (*A*/*l*)Δβ = (2*p*/l)Δβ, (4)

where *l* is the base pair length along the DNA axis, *p* is the persistent length. All hydrodynamic measurements were performed at a temperature of 21 °C.

### 3.4. Spectral Methods

UV absorption spectra of DNA were recorded using an SF-56 spectrophotometer (State Optical-Mechanical Factory, Saint Petersburg, Russia).

## 4. Conclusions

In this study, DNA conformation in alkaline and acidic solutions was examined. Polymer conformation in a solution is usually described considering certain parameters. The State Optical-Mechanical Plant persistent length is among the most important. However, its experimental determination is difficult since it is necessary to separate volume effects (polyelectrolyte swelling) and bending rigidity. The latter, along with the polymer hydrodynamic length, determines the unperturbed size of the macromolecule in an ideal solution. For double-stranded DNA, only the water solution is suitable. It is impossible to achieve an ideal solution in this case. Flow birefringence used in our research is one of the methods that allows the persistent length of DNA to be directly determined.

The DNA molecule in alkaline and acidic solutions changes its charge density. It was shown that protonation and deprotonation of DNA nitrogen bases within the double helix primarily alter the persistence length of the macromolecule. The pK of DNA in acidic and alkaline pH regions shifts to higher values as ionic strength (NaCl concentration) decreases. The new charges appearing on the DNA bases (positive charges at low pH and additional negative charges at high pH areas) cannot be shielded by counterions from a solution, and therefore their appearance is responsible for the change in the persistent length of DNA. The decrease and increase in the polyelectrolyte swelling of macromolecule as a result of its protonation and deprotonation, respectively, are mainly determined by the change in its persistence length.

The stability of the double-helical conformation ensures the steadiness of the equilibrium rigidity of this macromolecule. Delocalization of charge on DNA bases as a result of their protonation and deprotonation causes weakening and even disruption of the system of hydrogen bonds between complementary bases without changing the stacking interactions of base pairs, which is reflected in the absorption spectra of DNA at different pH levels.

In this study, the persistence length of DNA and its change in alkaline and acidic pH conditions were determined using hydrodynamic methods. The spectral study allowed us to assess the stability of DNA secondary structure in solutions with different pH. It was shown that the appearance of additional negative or positive charges on DNA bases, without destroying the double-stranded structure, causes a change in DNA persistence length.

## Data Availability

The original contributions presented in this study are included in the article. Further inquiries can be directed to the corresponding author(s).
